# Association of torque teno virus viremia with liver fibrosis in the first year after liver transplantation

**DOI:** 10.3389/fimmu.2023.1215868

**Published:** 2023-07-18

**Authors:** Bastian Engel, Irene Görzer, Alejandro Campos-Murguia, Björn Hartleben, Elisabeth Puchhammer-Stöckl, Elmar Jaeckel, Richard Taubert

**Affiliations:** ^1^ Department of Gastroenterology, Hepatology, Infectious Diseases and Endocrinology, Hannover Medical School, Hannover, Germany; ^2^ Center for Virology, Medical University of Vienna, Vienna, Austria; ^3^ Institute for Pathology, Hannover Medical School, Hannover, Germany

**Keywords:** torque teno (TT) virus, immunosuppression, biomarker, non-invasive test, personalized immunosuppression, individualization

## Abstract

**Introduction:**

Torque teno virus (TTV) replication is controlled by immune status, mirroring a degree of immunosuppression after solid organ transplantation. TTV viraemia (TTVv) was associated with acute cellular rejection and infection within the first year after liver transplantation (LT). Long-term data on TTV after LT and correlation with graft injury from protocol biopsies are limited.

**Methods:**

One hundred plasma samples paired with graft biopsies from a prospective single-center biorepository were analyzed.

**Results:**

The median time post-LT was 23 months (range, 2–298). TTVv was detectable in 97%. TTVv decreased over time after LT and showed a significant decline from year 1 to later time points. Hence, TTVv correlated negatively with histologic liver fibrosis (liver allograft fibrosis and Ishak scores) and positively with the overall immunosuppression degree quantified by an immunosuppression score in the first year after LT. There was no association with dosages or trough levels of single immunosuppressants. The pharmacodynamic marker TTVv did not correlate with pharmacokinetic assessments of immunosuppression degree [calcineurin inhibitor (CNI) trough levels or immunosuppressant dosages]—our clinical gold standards to guide immunosuppressive therapy. TTVv was independently associated with histologically proven liver fibrosis after LT in the first year after LT in multivariate analysis

**Discussion:**

The independent association of histological graft fibrosis with lower TTVv in year 1 underscores that a pharmacodynamic marker would be preferable to individualize immunosuppression after LT. However, a high variability of TTVv at the low immunosuppression doses given after the first year precludes TTV as a clinically useful marker after LT in the long-term liver transplant recipients.

## 1 Introduction

Despite continuous improvements in the survival rates in the first year after liver transplantation (LT), the long-term survival after the first year has not improved for decades. Causes of death after the first year following LT are much more related to long-term immunosuppression (IS) use (malignancies, infection, and cardiovascular death) than to graft loss itself (1, 2). In addition, the rates of chronic kidney injury are higher after LT compared with that of other solid organ transplants (3). In contrast to other solid organs, the prognostic relevance of acute rejection episodes in the first year after LT for long-term survival is limited, and the rates of chronic rejection are fortunately low (4). Therefore, attempts to individualize and minimize IS after LT are relatively safe compared to other solid organ transplants (5–7).

Another unique aspect of post-LT monitoring is the wide availability of inexpensive organ-specific markers of liver inflammation such as transaminases. Although transaminases are excellent for detecting major graft injury, such as acute rejection episodes, they are insensitive when it comes to detecting subclinical graft injuries (8). Such subclinical graft injury is important for individualizing IS to reduce its the unnecessary side effects such as renal damage (6, 9). Currently, IS after LT is guided by pharmacokinetic markers, such as trough levels or drug doses, tolerability, and markers of graft injury [liver enzymes, surveillance biopsies (svLBx), and liver stiffness measurements], but not by pharmocadynamic parameters of the immune system itself, as these are costly and hardly standardized.

Quantification of replication of the widespread and apathogenic torque teno virus (TTV) provides an indirect measure of the degree of IS (10). TTV replicates ubiquitously in the human body, and its replication is controlled by the immune system (10). TTV viraemia (TTVv) inversely correlates with the degree of IS. In the previous studies following various solid organ transplants, low TTV replication was associated with rejection and increased TTV replication with infection as a side effect of IS (10). The same has been shown in liver transplant recipients (LTRs) early after LT (11–14). Furthermore, TTV replication has been associated with subclinical graft injury after kidney transplantation (15).

The aim of the current single-center retrospective analysis of the liver and blood samples from a prospectively collected biorepository after LT was to study the association of TTVv with clinical and subclinical graft injury and the degree of IS within and after the first year after LT.

## 2 Patients and methods

### 2.1 Patients

We included 100 samples of 80 adult LTRs without replicative viral hepatitis who underwent a liver graft biopsy (LBx) within the prospective LBx repository at our center between 2008 and 2019. LTRs were included if they provided a written informed consent and had a plasma sample stored for research use. LT took place between 1990 and 2017. Both patients with stable liver graft function [liver enzymes < 2× upper limit of normal (ULN)] that had svLBx and with clinical T-cell–mediated rejection (cTCMR) that had indication biopsy were included. Plasma samples were collected within 24 h of the liver biopsy and cryoconserved at −80°C.

The current study is based on our previously published cohort from our ongoing prospective LBx biorepository in LTR (16). There was not enough plasma left for some patients from the published cohort, which led to a reduction of the original cohort size. In addition, we also included all patients with available liver biopsy and paired plasma samples that provided a written informed consent and had a histopathological evaluation of graft injury other than cTCMR and subTCMR (IND).

The study was approved by the local Ethics Committee (protocol number 933 for project Z2 of comprehensive research center 738; MHH Ethikkomission, Hannover, Germany). The study was conducted according to the ethical guidelines of the Declarations of Helsinki and Istanbul.

### 2.2 Biochemical measurements

Standard laboratory parameters were derived from patients’ records and were initially measured in clinical routine using high throughput techniques.

Donor-specific antibodies (DSAs) were detected as described recently (17). The incidence of DSA positivity was analyzed using a mean fluorescence intensity threshold of ≥ 1,000.

### 2.3 Immunosuppression scoring

For the assessment of the overall degree of IS, we calculated a semi-quantitative score as previously published by Vasudev et al. (18) for the kidney transplant recipients and utilized by our group recently in LTRs (6, 19). One point was assigned to each of the following drug dosages: tacrolimus (TAC) of 2 mg, cyclosporine (CsA) of 100 mg, mycophenolate mofetil (MMF) of 500 mg, prednisolone of 5 mg, and azathioprine of 100 mg. In addition, we assigned one score point to 1.5 mg of everolimus (EVR) or sirolimus (SIR) as previously published (6, 19).

### 2.4 Liver biopsies and histological grading and staging

Liver biopsies were performed percutaneously with local anesthesia. The biopsy cylinder was fixed in 4% neutral-buffered formalin and embedded in paraffin wax.

Histological examination and scoring for the rejection activity index (RAI) (20), inflammation grade and fibrosis stage (Ishak score) (21), central perivenulitis, portal microvasculitis, ductular reaction (22), fatty liver disease (23), and total liver allograft fibrosis (LAF) score (24) were performed by experienced liver pathologists in a blinded fashion. At least moderate fibrosis was defined as periportal fibrosis (Ishak F) ≥ 2 and/or any LAF score component ≥ 2. SubTCMR and cTCMR were defined as recently published (16). In detail, subTCMR was defined by a Banff RAI ≥ 1 + 1 + 1, and the patients had to have transaminases [aspartate aminotransferase (AST) and alanine aminotransferase (ALT)] and alkaline phosphatase (AP) below two times ULN. Gamma-glutamyl transferase (gGT) had to be stable or declining prior to svLBX, even if elevated above two times ULN, to be classified as subTCMR. cTCMR was defined as Banff RAI ≥ 1 + 1 + 1, and the presence of elevated liver enzymes (AST, ALT, and/or AP) above two times ULN. Biopsies of patients with any evidence of disease recurrence, active viral infection (e.g., viral hepatitis), or bacterial infections were excluded. No histological signs of rejection (NHR) was defined as RAI ≤ 1. There was no relevant inflammation and no relevant fibrosis (Ishak F ≤ 1, each LAF score component ≤ 1) in samples with NHR, and liver enzymes were within the normal range.

### 2.5 TTV DNA quantitation

Total nucleic acid was extracted from 200 µL of plasma using the NucliSENS easyMAG platform (bioMerieux, France) as recommended by the manufacturer and eluted in 50 µL of an elution buffer. TTV DNA quantitation was performed by an in-house TaqMan real-time PCR using forward primer AMTS: 5′-GTGCCGIAGGTGAGTTTA-3′, reverse primer AMTAS: 5′-AGCCCGGCCAGTCC-3′, and probe AMTPTU: 5′FAM-TCAAGGGGCAATTCGGGCT-3′TAMRA as described previously (25). The highly conserved untranslated region upstream of Open reading frame 1 (ORF1) is the real-time PCR target site allowing detection of all known TTV species (26). The quantitative PCR reactions were performed in a volume of 25 µL using 2× TaqMan Universal PCR Master Mix (Applied Biosystems, Foster City, CA, USA), containing 5 µL of extracted DNA, 400 nM of each primer, and 80 nM of the probe. Thermal cycling was started for 3 min at 50°C, followed by 10 min at 95°C, and then by 45 cycles at 95°C for 15s, at 55°C for 30s, and at 72°C for 30s using the 7300 Real Time PCR System (Applied Biosystems, Foster City, CA, USA). The linear range of TTV quantitation ranges from 2.7 to 10.7 log_10_ copies/mL as determined using 10-fold dilutions of a plasmid standard. The limit of detection in plasma is 2.7 log_10_ copies/mL. In each run, positive and negative controls were included. Prior to the extraction, 5 µL of phocine herpesvirus (PhHV) DNA as internal spike-in control was added to all plasma samples. None of the samples showed any signs of PCR inhibition as assessed by quantitation of PhHV DNA (cycle threshold, 30 ± 2.0).

### 2.6 Statistics

Statistical analyses were performed with SPSS (version 27, SPSS, Inc., Chicago, IL), R Statistical Software (version 4.1.2, R Core Team), and GraphPad Prism (version 9.4.0, GraphPad Software Inc., La Jolla, CA). The Mann–Whitney U-test was used to compare non-parametric continuous variables between two independent groups. The Kruskal–Wallis test was used for the comparison of non-parametric continuous variables between more than two independent groups. Adjustment for multiple comparisons was performed with Dunn’s *post-hoc* test. The Fisher’s exact test was used to compare categorical variables between two independent groups. Spearman’s rank correlation coefficient was used for correlation analysis and was plotted using the corrplot package (27) in R Statistical Software. Multivariate linear regression analysis was performed using the Tidyverse (28) package in R Statistical Software. Variables for multivariate linear regression were selected depending on their significant differences in univariate analysis and/or their causal relation to TTVv in the literature. $f^{2}$ was calculated as follows and published by Cohen (29): $f^{2}=\frac{R^{2}}{1-R^{2}}$ . P-values below 0.05 (two-tailed) were considered statistically significant in all analyses.

## 3 Results

### 3.1 Baseline demographics and cohort description

Of all 100 samples analyzed, TTV was detectable in the plasma in 97%. Three patients without detectable TTVv were excluded from further analyses. The characteristics of the cohort are summarized in **Table 1**. Seventy-five patients had svLbx, whereas 22 patients were indicated for biopsy due to elevated liver enzymes (AST, ALT, and/or AP ≥ 2× ULN). Only one patient had detectable cytomegalovirus (CMV) replication, which precluded further analysis between CMV and TTVv (not shown). TTVv did not correlate with age (Spearman’s Rho: 0.05; p = 0.66, n = 97) and did not differ between male and female LTR [median log_10_ TTV copies/mL (range), 5.9 (2.5–9.4) vs. 6.0 (3.2–8.2); Mann–Whitney U-test, p = 0.63). Of note, TTV replication did not correlate with any laboratory tests for liver injury (AST, ALT, AP, gGT, and bilirubin) (**Supplementary Figure 1**). TTVv was lower in patients with autoimmune liver diseases as cause of transplantation when compared to all other liver diseases (**Supplementary Figure 2A**), mainly due to a non-significant trend of lower TTVv in patients with autoimmune liver diseases being transplanted more than 1 year before sampling (**Supplementary Figure 2B**). A more detailed analysis comparing all liver diseases against each other revealed no significant differences (**Supplementary Figures 2C, D**).

**Table 1:** Patient and clinical data.

General information	
Patient number	97
TTV (log_10_) (copies/mL) [median (range)]	5.9 (2.5–9.4)
Age (years) [median (range)]	51 (18–69)
Female sex [n (%)]	35 (36.1)
Time after LT (months) [median (range)]	17 (2–298)
Surveillance LBx [n (%)]	75 (77.3)
Indication LBx [n (%)]	22 (22.7)
Laboratory parameters	
AST (times upper limit of normal) [median (range)]	0.8 (0.3–7.2)
ALT (times upper limit of normal) [median (range)]	0.6 (0.1–11.8)
AP (times upper limit of normal) [median (range)]	0.9 (0.2–8.0)
gGT (times upper limit of normal) [median (range)]	1.4 (0.2–38.7)
Bilirubin (times upper limit of normal) [median (range)]	0.6 (0.2–4.3)
Platelets (/nl) [median (range)]	177 (43–656)
Creatinine (µmol/l) [median (range)]	95 (57–792)
DSA positive [n (%)]	43 (44.3)
Histopathological characteristics	
cTCMR [n (%)]	13 (13.4)
subTCMR [n (%)]	39 (40.2)
IND [n (%)]	26 (26.8)
NHR [n (%)]	19 (19.6)
RAI [median (range)]	3 (0–8)
mHAI [median (range)]	3 (0–9)
Ishak fibrosis stage [median (range)]	1 (0–6)
LAF score [median (range)]	1 (0–8) (n = 96)
Reason for LT	
AILD [n (%)]	32 (33)
Alcoholic [n (%)]	20 (20.6)
HCC [n (%)]	12 (12.4)
Viral [n (%)]	4 (4.1)
Cryptogenic [n (%)]	12 (12.4)
Other [n (%)]	17 (17.5)
Immunosuppression	
Mono-IS [n (%)]	3 (3.1)
Dual-IS [n (%)]	25 (25.8)
Triple-IS [n (%)]	69 (71.1)
TAC [n (%)]	42 (43.3)
CsA [n (%)]	51 (52.6)
EVR [n (%)]	2 (2.1)
SIR [n (%)]	2 (2.1)
IS score [median (range)]	5 (1.8–12.0)

TTV, torque teno virus; AST, aspartate aminotransferase; ALT, alanine aminotransferase; AP, alkaline phosphatase; gGT, gamma-glutamyltransferase; cTCMR, clinically overt T-cell mediated rejection; subTCMR, subclinial TCMR; IND, indeterminate graft injury other than cTCMR and subTCMR; NHR, no histological signs of rejection; RAI, rejection activity index; mHAI, modified histological activity index according to Ishak et al. (21); LAF score, liver allograft fibrosis score according to Venturi et al. (24); DSA, donor-specific anti-HLA antibodies; LT, liver transplantation; CLD, chronic liver disease; AILD, autoimmune liver disease; HCC, hepatocellular carcinoma; IS, immunosuppression; TAC, tacrolimus; CsA, cyclosporine A; EVR, everolimus; SIR, sirolimus; IS score, immunosuppression score.

### 3.2 TTV levels are dependent on time after LT

**Figure 1:**
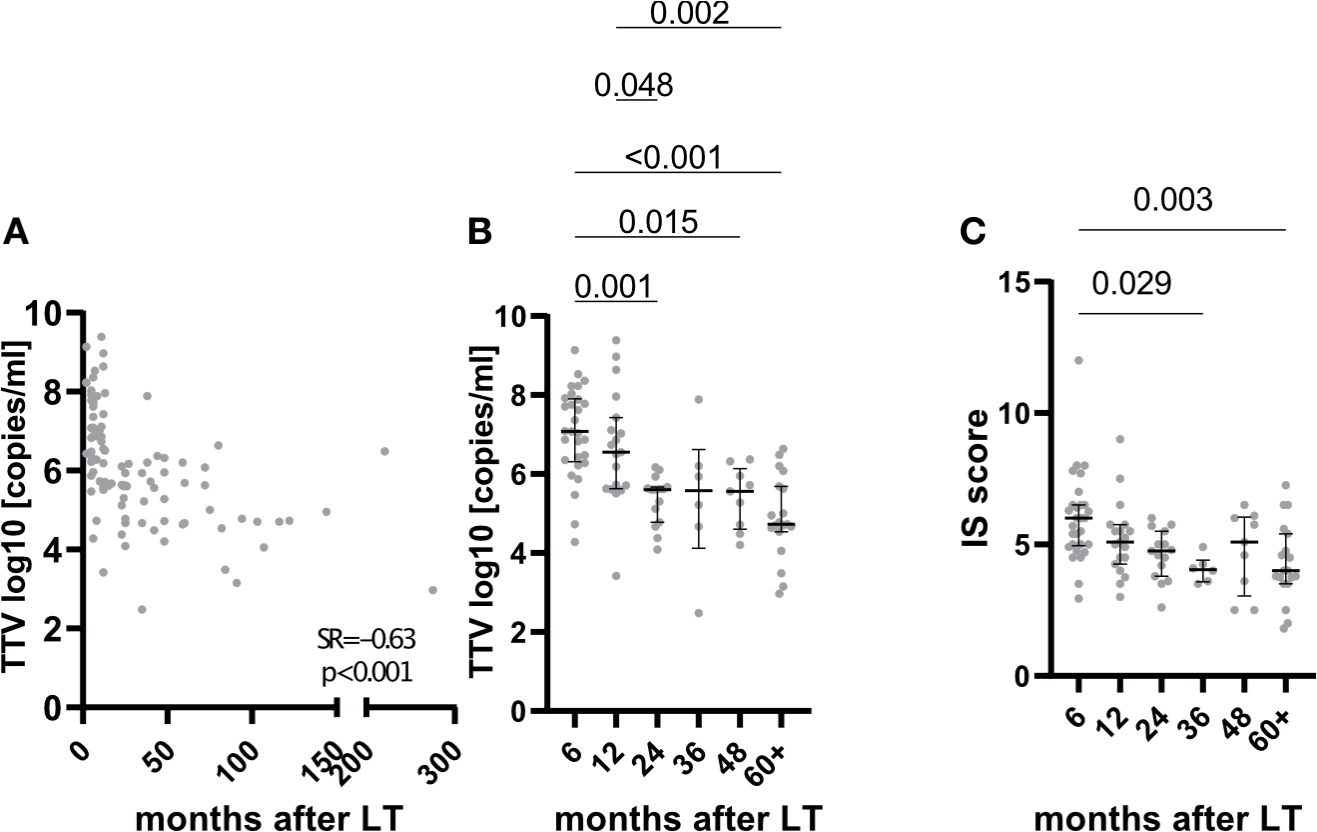
Association of TTV with time after transplantation. TTVv correlated with time after LT both linear **(A)** and if grouped **(B)**. Degree of immunosuppression (IS) as quantified by IS score correlated with time after LT **(C)**. For panels **(B)** and **(C)**, grouping was as follows: 6, patients with liver biopsy (LBx) in the first 6 months after liver transplantation (LT); 12, LBx between 6 and 12 months after LT; 24, LBx in the second year after LT; 36, LBx in the third year after LT; 48, LBx in the fourth year after LT; 60+, LBx 60 months and later after LT. Spearman rank correlation coefficient (SR) with its respective p values is outlined **(A)**. Median and Interquartile range are shown for categorical variables **(B, C)**. Kruskal–Wallis test with Dunn’s *post-hoc* Test was used for comparison between more than two categorical variables.

TTV levels are known to decline after solid organ transplantation, even within the first year (11, 13). Hence, the correlation between TTVv and time after transplantation was analyzed. TTV replication showed a negative correlation with time post-LT (Spearman’s Rho: −0.63, p < 0.001, n = 97; **Figure 1A**) and a decrease from the first year to later time points (**Figure 1B**). However, the degree of IS, as measured by the IS score, decreased correspondingly with time after transplantation (**Figure 1C**). This decrease in TTVv and IS score over time was observed not only in the overall analysis but also longitudinally in directly paired samples (**Supplementary Figure 3A**). The decrease of TTV replication in these patients was accompanied by a decreased degree of IS (**Supplementary Figure 3B**). As a marked decline of TTVv was seen beyond the first year after LT, we stratified patients according to their time after LT for further analyses. The first group includes patients within the first year after LT, and the second group includes patients beyond the first year after LT. Patients after the first year after LT had lower TTVv and IS score and higher modified histological activity index (mHAI), Ishak fibrosis stage, and LAF score (**Supplementary Table 1**).

### 3.3 Relationship between TTV viraemia and liver graft injury

**Figure 2:**
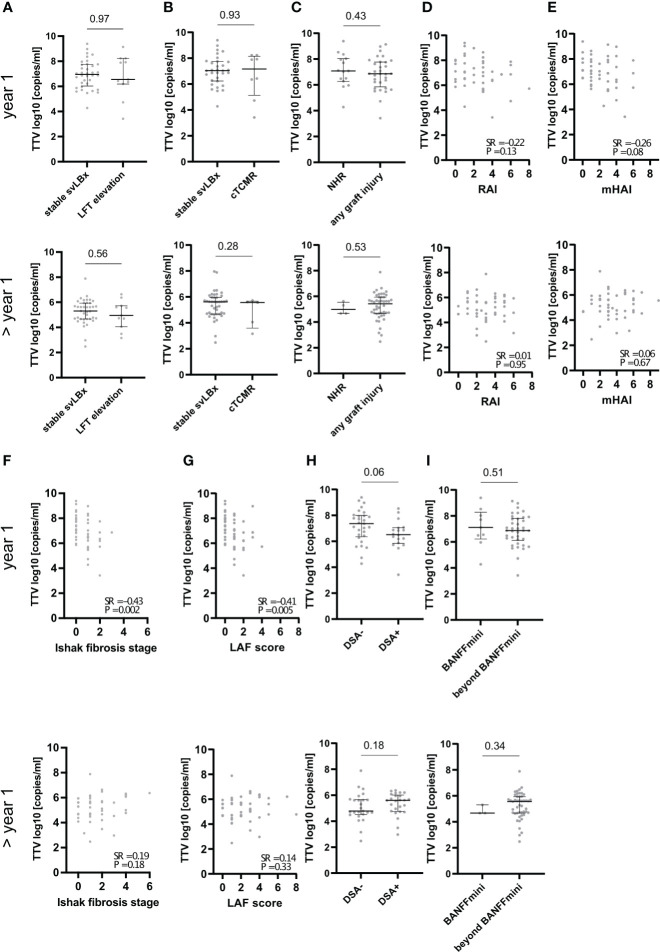
Association of TTV with graft injury and donor-specific antibodies depending on time after LT. The respective upper panels show data from patients that had sampling within 1 year after LT (indicated by “year 1” on the left side), and the lower panels show data from patients that had sampling later than 1 year after LT (indicated by “> year 1” on the left side). TTVv was not different between patients without elevated liver function tests< 2× ULN (stable svLBx) and with elevated liver function tests (LFT elevation) **(A)** or patients with clinically overt T-cell–mediated rejection (cTCMR) **(B)**. TTVv was different between patients with no histological signs of rejection (NHR) and those with any graft injury **(C)**. TTVv correlated with Ishak fibrosis stage and liver allograft fibrosis (LAF) score in the first year after LT but not with rejection activity index (RAI) and mHAI and not with any score later than 1 year after LT **(D–G)**. TTVv was not different dependent on the presence of donor-specific antibodies (DSA) **(H)**. TTVv was not different dependent on the fulfillment of BANFF criteria for the reduction of immunosuppression (BANFFmini) **(I)**. Spearman rank correlation coefficient (SR) with its respective p values is outlined **(D–G)**. Median and Interquartile range (IQR) are shown for categorical variables **(A–C, H, I)**. Mann–Whitney U-test was used for comparison between two categorical variables.

As a correlation between low TTV replication and graft injury by cTCMR within the first year after LT has been described in the literature (11), we stratified patients into two groups. The first group consisted of LTR with stable graft function with AST, ALT, and AP < 2× ULN. These patients received a liver biopsy for histological monitoring (svLBx) according to our local protocol (6). The other group consisted of patients with clinical graft hepatitis (elevation of AST, ALT, and/or AP above 2× ULN). There were no differences in TTV replication between patients with svLBx and those with clinically evident graft hepatitis (liver enzymes ≥ 2× ULN), regardless of the findings in the indication biopsy (**Figure 2A**, **Supplementary Figure 4A**). Likewise, TTVv was not significantly lower in cTCMR compared with svLBx, neither in the first year nor thereafter, or in the overall cohort (**Figure 2B**, **Supplementary Figure 4B**). Clinically overt TCMR occurred at a median of 8.4 months after LT (range, 2–107 months; n = 13).

TTVv was not different in patients with any histological graft injury compared to patients with no histological signs of rejection (**Figure 2C**). However, in the overall cohort that was not stratified according to time after LT, TTVv was lower in patients with any histological graft injury (**Supplementary Figure 4C**). Next, the correlation of TTVv with different histological scores of graft injury was assessed according to time after LT. TTVv did not correlate with histological scores of liver inflammation and rejection when assessed separately within or after the first year after LT (**Figures 2D, E**, **Supplementary Figure 4D**) but correlated negatively with mHAI in the overall cohort (**Supplementary Figure 4E**). TTVv correlated negatively with histological staging of graft fibrosis (Ishak fibrosis stage and LAF score) in the first year after LT (**Figures 2F, G**) but not at later time points. The correlation of TTVv with fibrosis remained when the overall cohort was assessed (**Supplementary Figures 4F, G**). In addition, the mere presence or absence of DSA as marker of allosensitization was not associated with TTV replication (**Figure 2H**, **Supplementary Figure 4H**). TTVv did not predict whether patients met Banff histological criteria for reduction of IS irrespective of the time after LT (**Figure 2I**, **Supplementary Figure 4I**). Neither the presence of graft injury (87.5% vs. 76.9%, p = 0.28) nor the median mHAI (3 vs. 3, p = 0.87), LAF score (1 vs. 1, p = 0.59), or Ishak fibrosis stage (1 vs. 1, p = 0.88) was different in patients with autoimmune liver disease prior to LT compared to any other etiology of pre-LT liver disease. Graft injury was more frequent and pronounced in patients beyond the first year after LT (92.0% vs. 68.1%, p = 0.004), which had higher median mHAI (3 vs. 2, p = 0.002) and LAF score (2 vs. 1, p < 0.001) (**Supplementary Table 1**). In conclusion, graft fibrosis was more pronounced in patients with lower TTV replication, suggesting a relationship between both parameters and lower IS, but only in the first year after LT.

### 3.4 No association between routine pharmacokinetic markers of immunosuppression and graft injury

Next, the association between graft injury and measures of IS was analyzed. Unexpectedly, the classic measures of IS degree were not associated with histological graft injury. Neither TAC nor CsA trough levels correlated with mHAI, RAI, Ishak F, or LAF score in the first year after LT as well as in the overall cohort (**Table 2**, **Supplementary Table 2**). Histological scores of graft injury also did not differ between LTR with different daily MMF doses (0 g of MMF vs. 0–1 g of MMF vs. > 1 g of MMF) (**Supplementary Figures 5A–D**). Conversely, the median RAI was higher in patients with prednisolone [3 (range, 0–8)] than that in patients without prednisolone [1 (range, 0–6); p = 0.02] (**Supplementary Figure 4F**). The median mHAI, Ishak F, and LAF score did not differ depending on prednisolone intake (**Supplementary Figures 5E, G, H**). However, a semi-quantitative score assessing the overall degree of IS (18, 19) was negatively associated with histological scores for liver allograft fibrosis [LAF score (Spearman’s Rho: −0.34; p = 0.02)] in the first year but not thereafter (**Table 2**, **Supplementary Table 2**). On the contrary, TTVv showed a moderate correlation with both Ishak fibrosis stage and LAF score in the first year after LT as well as in the overall cohort (**Table 2**, **Supplementary Table 2**). Beyond the first year after LT, neither CsA trough levels nor the IS score or TTVv correlated with histological markers of graft injury (**Table 2**).

**Table 2:** Correlation of pharmacokinetic markers of immunosuppression degree with histological scores of graft injury.

Year 1				
	mHAI	RAI	Ishak F	LAF score
IS score	SR = −0.07 (p = 0.66, n = 47)	SR = −0.13 (p = 0.38, n = 47)	SR = −0.27 (p = 0.07, n = 47)	SR = −0.34 (p = 0.02, n = 47)
TAC trough level (µg/L)	SR = −0.004 (p = 0.99, n = 22)	SR = −0.10 (p = 0.68, n = 22)	SR = 0.01 (p = 0.98, n = 22)	SR = −0.07 (p = 0.77, n = 22)
CsA trough level (µg/L)	SR = 0.18 (p = 0.41, n = 23)	SR = −0.09 (p = 0.68, n = 23)	SR = −0.11 (p = 0.60, n = 23)	SR = −0.14 (p = 0.53, n = 23)
> year 1				
	mHAI	RAI	Ishak F	LAF score
IS score	SR = −0.16 (p = 0.28, n = 50)	SR = −0.07 (p = 0.62, n = 50)	SR = −0.25 (p = 0.08, n =50)	SR = −0.21 (p = 0.14, n = 49)
TAC trough level (µg/L)	SR = 0.33 (p = 0.14, n = 21)	SR = 0.03 (p = 0.91, n = 21)	SR = 0.62 (p = 0.003, n = 21)	SR = 0.62 (p = 0.003, n = 21)
CsA trough level (µg/L)	SR = −0.30 (p = 0.12, n = 28)	SR = −0.16 (p = 0.42, n = 28)	SR = −0.09 (p = 0.63, n = 28)	SR = −0.04 (p = 0.85, n = 27)

Correlation matrix between markers to quantify degree of immunosuppression (IS score, TAC trough level, and CsA trough level) and histological parameters of graft injury (mHAI, RAI, Ishak F, and LAF score). Values are provided as Spearman correlation coefficient (SR). P-value and number (n) are shown in brackets. RAI, rejection activity index; mHAI, modified histological activity index according to Ishak et al. (21); Ishak F, fibrosis staging according to Ishak et al. (21); LAF score, liver allograft fibrosis score according to Venturi et al. (24); TAC, tacrolimus; CsA, cyclosporine A.

### 3.5 Relationship between TTV viraemia and pharmacokinetic measures of immunosuppression

**Figure 3:**
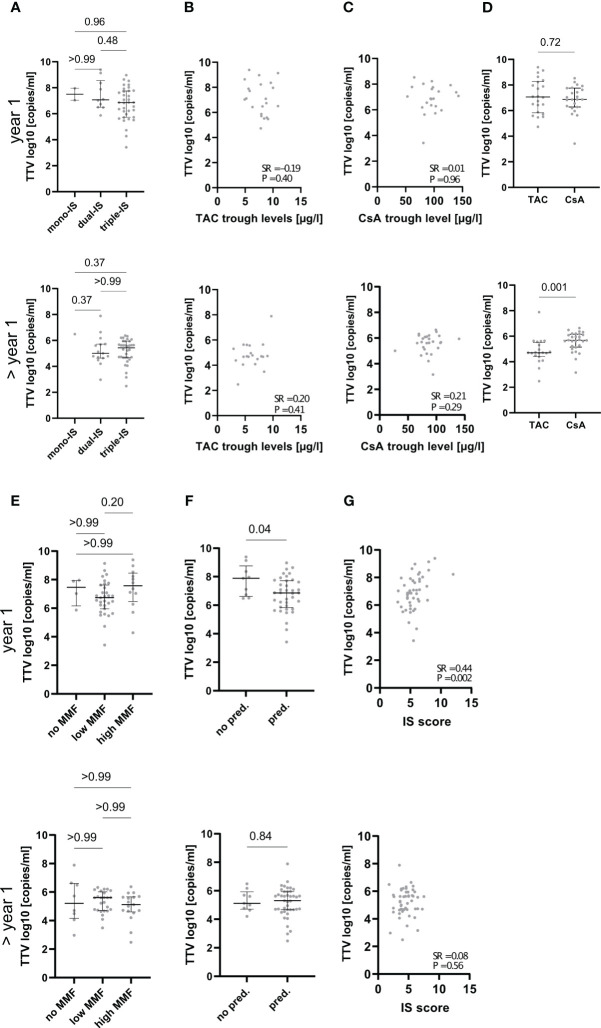
Association of TTVv with degree and type of immunosuppression. The respective upper panels show data from patients that had sampling within 1 year after LT (indicated by “year 1” on the left side), and the lower panels show data from patients that had sampling later than 1 year after LT (indicated by “> year 1” on the left side). **(A)** Magnitude of TTVv is not different dependent on the number of immunosuppressive drugs used. Mono/dual/triple-immunosuppression (IS): one, two, or three immunosuppressive drugs used, respectively. TTVv does neither correlate with tacrolimus (TAC) **(B)** or cyclosporine A (CsA) **(C)** trough levels. Patient stratification based on the primary immunosuppressive agent used demonstrates lower TTVv in patient on TAC later than 1 year after LT but not in the first year **(D)**. TTVv does not correlate with dosage of mycophenolate mofetil (MMF) (low MMF ≤ 1 g/day; high MMF > 1 g/day) **(E)**. TTVv does not correlate with usage of prednisolone (pred.) **(F)**. TTVv correlates with IS score in the first year after LT but not thereafter **(G)**. Spearman rank correlation coefficient (SR) with its respective p values is outlined **(B, C, G)**. Median and IQR are shown for categorical variables **(A, D, E, F)**. Kruskal–Wallis test with Dunn’s *post-hoc* Test was used for comparison between more than two categorical variables, and Mann–Whitney U-test was used for comparison between two categorical variables.

Next, we investigated whether TTVv correlated with the overall degree of IS. TTVv did not differ between patients with mono-, dual-, or triple-IS (**Figure 3A**, **Supplementary Figure 6A**). Regardless of the choice of primary immunosuppressant, TTVv did not correlate with TAC or CsA trough levels, both in the overall cohort and in the subcohorts stratified according to time after LT (**Figures 3B, C**, **Supplementary Figures 6B, C**). Thus, there was no significant association between the pharmacokinetic and pharmacodynamic effects of the CNIs. In addition, TTVv did not differ significantly between LTR with CsA and TAC as the primary immunosuppressant in the overall cohort (**Supplementary Figure 6D**) but was lower in patients beyond the first year after LT on TAC as compared to CsA (**Figure 3D**). To assess the dependence of TTVv on MMF dose, patients were stratified into three MMF dose groups as mentioned earlier. No differences in TTVv were observed for MMF (**Figure 3E**, **Supplementary Figure 6E**. In the first year after LT, patients without prednisolone had higher TTVv, but this effect was not seen in the overall cohort or beyond the first year after LT (**Figure 3F**, **Supplementary Figure 6F**). TTVv was moderately correlated with IS score, with higher IS degree associated with higher TTV replication, but only in the first year after LT (SR = 0.44, p = 0.002) and not thereafter (Spearman’s Rho: 0.08, p = 0.56) (**Figure 3G**, **Supplementary Figure 6G**).

### 3.6 TTV viraemia is associated with histological graft injury independently from degree of immunosuppression in the first year after LT

Both the IS score and TTVv were associated with graft fibrosis in our cohort in the first year after LTx. To determine whether any of these two factors from the univariate analysis were independently associated with TTV replication, we performed a multivariate regression analysis of samples taken within the first year after LT. Although age and sex were not associated with TTVv in our cohort, both demographic parameters were included in the multivariate analysis because such associations have been reported in the literature (10). A multiple linear regression model was used to test whether TTVv, IS score, age, and sex significantly predicted LAF score as an LT-specific score of liver graft fibrosis, which demonstrated an association with both IS score and TTVv. The fitted regression model was follows: LAF score = 4.63 − 0.09*(IS score) − 0.35*[TTV (log_10_)] − 0.09*(male sex) − age*(0.02) (p = 0.02). The overall model significantly predicted LAF score. Only TTVv was significantly associated with the LAF score (ß = −0.35, p = 0.02), whereas age (ß = −0.02, p = 0.23), male sex (ß = −0.09, p = 0.77), and IS score (ß = −0.09, p = 0.39) did not. The effect size, quantified by, was 0.35, indicating a large effect size. TTVv only explained 26% of the variance in LAF score (multiple R-squared: 0.26, adjusted R-squared: 0.18) (**Table 3**).

**Table 3:** Multivariate linear regression model to predict LAF score by clinical parameters.

Coefficients	ß Estimate	Std. Error	T-value	P-value
**(Intercept)**	**4.63**	**1.08**	**4.28**	**< 0.01**
Age at Lb	−0.02	0.01	−1.22	0.23
Male Sex	−0.09	0.31	−0.30	0.77
**TTV (log_10_)**	**−0.35**	**0.14**	**−2.47**	**0.02**
IS score	−0.09	0.11	−0.87	0.39

Multivariate linear regression model: LAF score ~ Age at Lb + Male Sex + TTV (log_10_) + IS score.

TTV, torque teno virus; LT, liver transplantation; IS score, immunosuppression score.

## 4 Discussion

TTVv has been described as a marker of IS degree and thus for the risk of rejection in LTR, mainly in the first year after LT (12, 13), but lower levels have also been associated with graft hepatitis in pediatric LTR beyond the first year after LT (30). Whereas TTVv has been associated with cTCMR in the first year after LT (11), an association between TTVv and cTCMR in LTR both in the first year after LT and beyond could not be confirmed in this representative cross-sectional quantification of TTVv in the long-term follow-up after LT. The low level of IS in LTR compared to lung and kidney transplant patients, especially after the first year, may be responsible for this finding. Of note, the long-term magnitude of TTVv (log_10_ copies/mL) appears to be lower in LTR compared to kidney (31, 32) and lung transplant recipients (33) and is even close to the range of healthy blood donors (34) after the first year after LT.

However, our study adds to the existing literature as it demonstrates an association between TTVv and histological quantification of liver allograft fibrosis in graft biopsies, such that a greater graft injury as a possible consequence of “under”-IS was associated with lower TTVv because of better control of TTV replication by immune cells. The association of subclinical graft injury and expression of rejection associated transcripts with progression of graft fibrosis was recently demonstrated by another LT center (35). Interestingly, the association of TTVv with histological measures of graft fibrosis (LAF score) was more pronounced than with TCMR (RAI). This confirms our previous finding that progressive liver graft fibrosis was not stringently associated with TCMR features (8). Ultimately, the current results fuel the ongoing scientific debate on the histopathological criteria for late TCMR manifestations after LT.

Interestingly, liver graft injury was hardly associated with classical and clinically used pharmacokinetic measures such as CNI trough levels or simple doses of immunosuppressants, neither in the first year after LT nor thereafter. Similarly, TTV replication was not associated with these classical measures of IS degree, which has been heterogeneously assessed in previous studies on TTV in LTR (11–14, 30). Although these studies use different clinical classifications of immunosuppressive regimens (e.g., number of drugs or primary immunosuppressant) and graft injury (e.g., clinically apparent hepatitis or biopsy proven rejection) viral loads in already published studies, mainly described in the first year after LT, are comparable with those described in our study (11, 13, 30). We were not able to validate previous findings based on these classifications but were able to show an association of TTVv with histopathological assessment of graft fibrosis in the first year after LT. Although one study in pediatric LTR that included patients over several years after LT was able to demonstrate different median TTV levels in patients with chronic graft hepatitis compared to patients without graft hepatitis (30), we were not able to validate this findings in adult LTR in our study although the absolute TTV levels were in a comparable range in the previous study and ours (**Figure 2A**). Although we did not observe the previously published findings in our cohort, we add another layer of information by associating TTV quantitation with histopathological scoring systems. The association of liver graft injury and TTVv with an arbitrary semi-quantitative score summarizing individual immunosuppressant dosages suggests that the measures of total or cumulative IS degree may be more helpful in guiding the individual IS management (18, 19). However, in contrast to the IS score, quantification of TTV replication promises to provide information about individual “third”-party immunity in the donor-recipient immune interaction, just like other pharmacodynamic markers, e.g., the Immuknow^®^ assay (36). The IS guidance provided by this Immuknow^®^ assay was associated with an improvement in 1-year survival after LT through a reduction in infection-related deaths. However, TTVv showed substantial longitudinal changes and high variability, which prevents unselective cross-sectional use of the pharmacodynamic marker TTVv as non-invasive marker of liver graft injury. However, TTVv was different in patients with autoimmune liver diseases that led to LT, which are regarded to be more sensitive to alloimmune injury. Regarding “third”-party immunity, TTVv was not influenced by DSA status, the most widely available tool to assess the former in LTR. The non-availability of Human leukocyte antigen (HLA) crossmatch data in our LTR cohort, as LTRs are not matched by HLA status, prevented further insights into the connection between HLA mismatch and TTVv. Finally, TTVv was not a suitable marker for detecting relevant subclinical graft injury beyond the criteria justifying minimization of IS (BANFFmini) according to the most recent BANFF consensus document of 2016 (22) that were recently used to guide IS management in our center (6). The immunological niche of the liver, which allows for lower IS compared to other solid organ recipients, resulting in TTV levels in the range of healthy blood donors (34) and high inter-individual variation, may prevent uncritical use of TTVv to guide IS in LTR beyond the first year for which we demonstrate no association of TTVv with histopathological criteria of graft injury.

A unique strength of our study is the wide availability of svLBx as the gold standard for detecting graft injury within the first year after LT and beyond. In a prospective observational study, subclinical graft injury was also associated with subclinical alloreactivity in kidney transplant recipients (15). In this study, the number of days with a viral load below 6 log_10_ TTV copies/mL was associated with the risk of developing subclinical kidney graft injury. Time after transplantation as a potentially confounding covariate was balanced by including only samples from the same time point after kidney transplantation. In the broader context of solid organ transplantation, current evidence in kidney transplant recipients suggests a cutoff of > 6.6 log_10_ TTV copies/mL for increased infection risk and < 4.6 log_10_ TTV copies/mL for increased risk of rejection with the newly available Conformite Europene (CE)-certified PCR for quantification of TTV replication (10). This corresponds to cut-offs of > 8 log_10_ TTV copies/mL and < 6 log_10_ TTV copies/mL respectively, with the in-house assay used in our and most other previous studies on TTV (37). In lung transplant recipients, the cutoff values with the in-house assay were 7 and 9.5 log_10_ copies/mL for “optimal” levels of IS (10). In LTR, the data are less conclusive, but, given the immunological privilege of the liver and the possibility of spontaneous operational tolerance, the cutoff values are likely to be even lower than that in kidney transplant recipients and more in line with those measured in healthy individuals (34). Technically, the in-house and CE-certified assays correlate well, and future studies will certainly be conducted with the CE-certified assay, which offers manufacturer-guaranteed reproducibility for larger prospective studies as just recently assessed in kidney transplant recipients (37). However, these cutoff values provide a narrow therapeutic window of “optimal” levels of IS and proclaim the biological stability of TTV replication independent of inter-individual variation and intra-individual variation due to unknown factors as a prerequisite for TTV measurements to guide immunosuppressive therapy. Neither our study nor other studies have been able to provide insights into other potential biological determinants that could explain these variations. Furthermore, the majority of LTR in our study have TTV levels that are below the suggested cutoff value for increased risk of rejection in other solid organ transplantations, especially LTR after the first year whose TTV levels are in the range of healthy individuals (34). Especially regarding the marked decline of TTV levels beyond the first year after LT, which was also demonstrated in pediatric LTR (30), future studies are needed to determine not only organ-specific but also time-dependent cutoff values for increased risk of rejection and infection. The published cutoff values may therefore not readily translate to the daily clinical care of LTR, but future studies of long-term viral kinetics in LTR will need to be conducted to determine the ultimate clinical application of TTV measurements for risk stratification in these patients.

In addition to the strengths of our study, which provides insights into the long-term viral kinetics of TTV in LTR using a cohort-based approach and the association of TTV with graft injury in widely available, prospectively collected svLBx, it has some limitations. To date, our biorepository has only included serial sampling paired with liver biopsies, i.e., LTR had to have a reason to perform a liver biopsy during their clinical care for a blood sample to be stored in our biorepository, but there is no additional sampling, e.g., at routine visits in the outpatient clinic. As the biorepository includes patients after LT but not already on the waiting list, pre-LT samples are not available from individual patients before LT. Both limitations prevent further insights into viral kinetics of TTV, especially the identification of factors related to inter- and intra-individual variations in TTV levels in LTR. However, our cross-sectional approach more closely resembles routine clinical use. In addition, blood samples for TTV quantification were not collected during infections. In the lung and kidney transplant recipients, quantification of TTVv can be used to detect “over”-IS, as it has been able to predict infectious complications (10). Testing for viral infections is not routinely done as surveillance at our center but only in case of clinical suspicion, e.g., elevated liver function tests, and no routine testing is performed for infections with hepatotropic viruses that are known to decrease TTV load (30). Apart from the limitation in studying the association between TTV and infectious complications in our cohort related to the sampling protocol, only one patient had CMV reactivation, which prevents a solid conclusion on the usefulness of TTV quantification to predict infectious complications in the long-term in LTR. However, other studies also failed to demonstrate a clear association between higher TTVv and infections after LT (10).

In summary, non-invasive detection of mainly subclinical graft injury in LTR is necessary to prevent the development of fibrosis and its complications in the long-term. Detection of graft inflammation and fibrosis at an early stage is crucial for any intervention to halt or slow down the progression of fibrosis and its sequelae. The pharmacodynamic marker of TTV replication, despite its limitations, is associated with graft injury in the first year after LT, whereas the pharmacokinetic markers currently used to guide immunosuppressive treatment are not. However, the time-dependent decline in TTV levels and the missing association with histopathological evaluation in LTR beyond the first year after LT limits the diagnostic value of TTV quantitation for liver fibrosis in long-term cross-sectional monitoring after LT. Finally, this study highlights that pharmacodynamic markers may be better suited to monitor the liver graft for (subclinical) injury, whereas the currently used non-invasive markers of IS degree or liver injury (9) fail to do so.

## Data availability statement

The raw data supporting the conclusions of this article will be made available by the authors, without undue reservation.

## Ethics statement

The studies involving human participants were reviewed and approved by MHH Ethikkommission, Hannover, Germany. The patients/participants provided their written informed consent to participate in this study. The study was conducted according to the ethical guidelines of the Declarations of Helsinki and Istanbul.

## Author contributions

Study concept and design: BE, EJ, and RT. Acquisition of data: BE, IG, and BH. Analysis and interpretation of data: BE, IG, AC-M, EJ, and RT. Drafting of the manuscript: BE and RT. Critical revision of the manuscript for important intellectual content: IG, AC-M, BH, EP-S, and EJ. Statistical analysis: BE, AC-M, and RT. Obtained funding: BE, EJ, and RT. Administrative, technical, or material support: BE, IG, AC-M, BH, EP-S, EJ, and RT. Study supervision: EP-S, EJ, and RT. All authors contributed to the article and approved the submitted version.
