# Supplementary material for: Association of torque teno virus viremia with liver fibrosis in the first year after liver transplantation
Source: Front Immunol. 2023 Jul 18;14:1215868. doi: 10.3389/fimmu.2023.1215868 (PMC10392936; doi:10.3389/fimmu.2023.1215868)
Supplement: Supplementary file 1 [file DataSheet_1.pdf]

Association of Torque Teno virus viremia with liver fibrosis in the first year after liver transplantation

Bastian Engel[1], Irene Görzer[2], Alejandro Campos-Murguia[1], Björn Hartleben[3], Elisabeth Puchammer-Stöckl[2], *Elmar Jaeckel[1, #], *Richard Taubert[1]

* authors contributed equally

Supplementary Material

<u>Supplementary figures</u>

Supplemental Figure 1

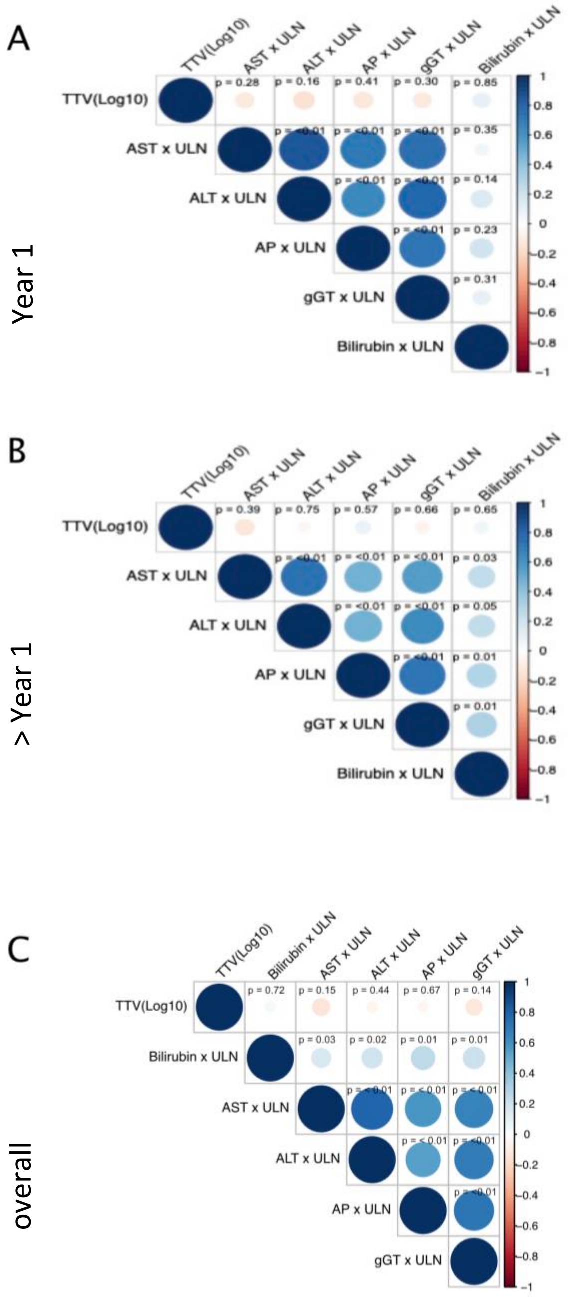

Suppl. Figure 1: Spearman correlation analysis of TTV replication with transaminases and cholestasis parameters as markers of liver injury

Spearman correlation of TTVv log10 transformed with non-invasive marker of liver injury in patients within the first year after LT (A), after the first year after LT (B) and in the overall cohort (C). Color indicates spearman correlation coefficient according to the accompanying scale. Size of the circles indicates p-values with larger circles indicating smaller p-values.

Supplemental Figure 2

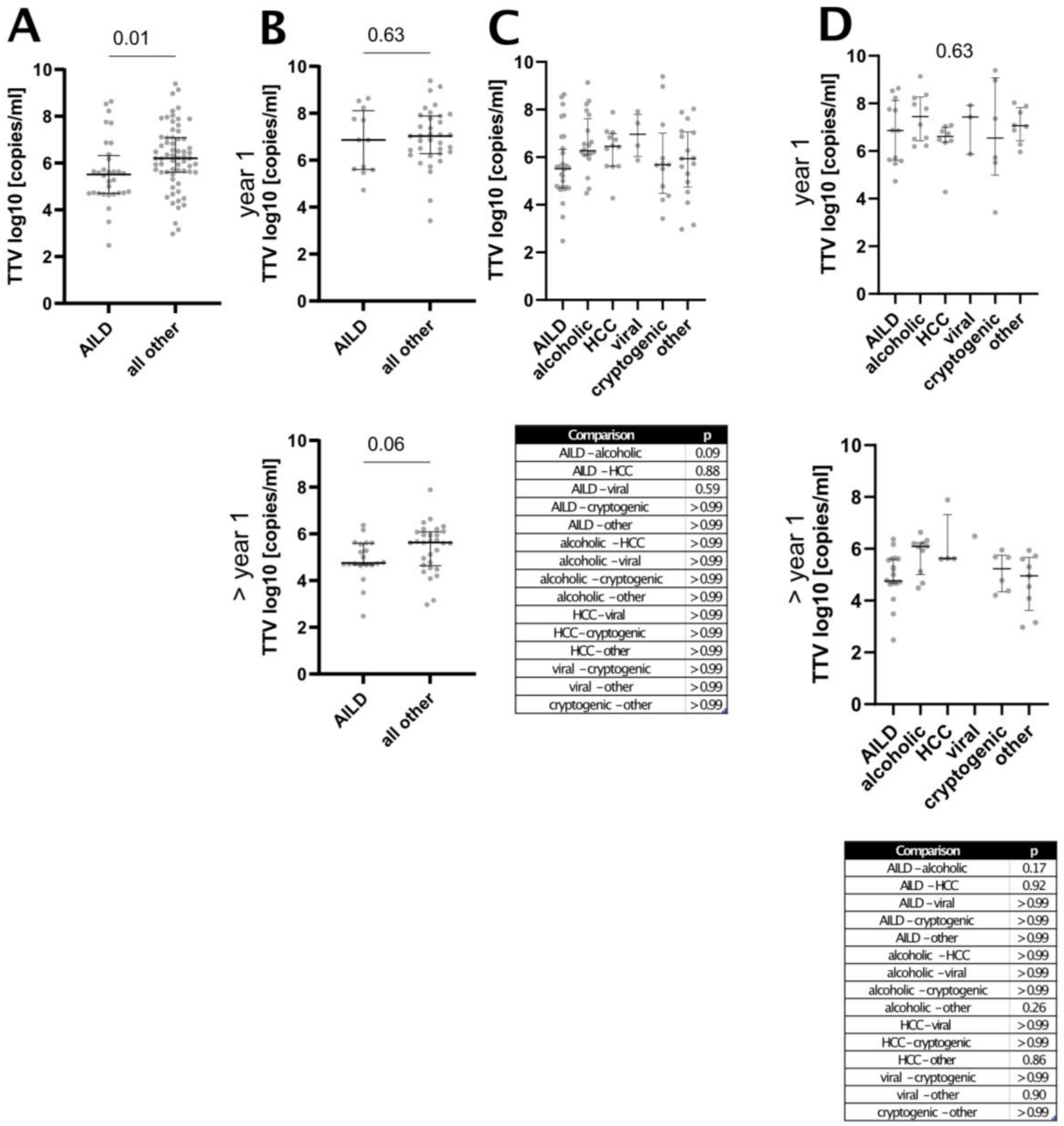

Comparison	p
AILD – alcoholic	0.09
AILD – HCC	0.88
AILD – viral	0.59
AILD – cryptogenic	>0.99
AILD – other	>0.99
alcoholic – HCC	>0.99
alcoholic – viral	>0.99
alcoholic – cryptogenic	>0.99
alcoholic – other	>0.99
HCC – viral	>0.99
HCC – cryptogenic	>0.99
HCC – other	>0.99
viral – cryptogenic	>0.99
viral – other	>0.99
cryptogenic – other	>0.99

Comparison	p
AILD – alcoholic	0.17
AILD – HCC	0.92
AILD – viral	>0.99
AILD – cryptogenic	>0.99
AILD – other	>0.99
alcoholic – HCC	>0.99
alcoholic – viral	>0.99
alcoholic – cryptogenic	>0.99
alcoholic – other	0.26
HCC – viral	>0.99
HCC – cryptogenic	>0.99
HCC – other	0.86
viral – cryptogenic	>0.99
viral – other	0.90
cryptogenic – other	>0.99

Suppl. Figure 2: Association of TTVv with liver disease leading to LT

TTV levels are compared between patients with autoimmune liver diseases (AILD) leading to LT and other etiologies, both in the overall cohort (A) and in patients with biopsy within the first year after LT and thereafter (B). Other etiologies are substratified in the overall cohort (C) and the sub-cohorts according to time after LT (D). Median and IQR are shown. Mann-Whitney-U test was used for two group comparisons and Kruskal-Wallis Test with Dunn's post hoc test for comparisons between more than two groups. The first panel in 2D spares group

comparisons as the Kruskal Wallis Test was not significant. The tables below 2C and the second graph in 2D show the two-group comparisons for the respective graph above for better readability of the graph.

Supplemental Figure 3

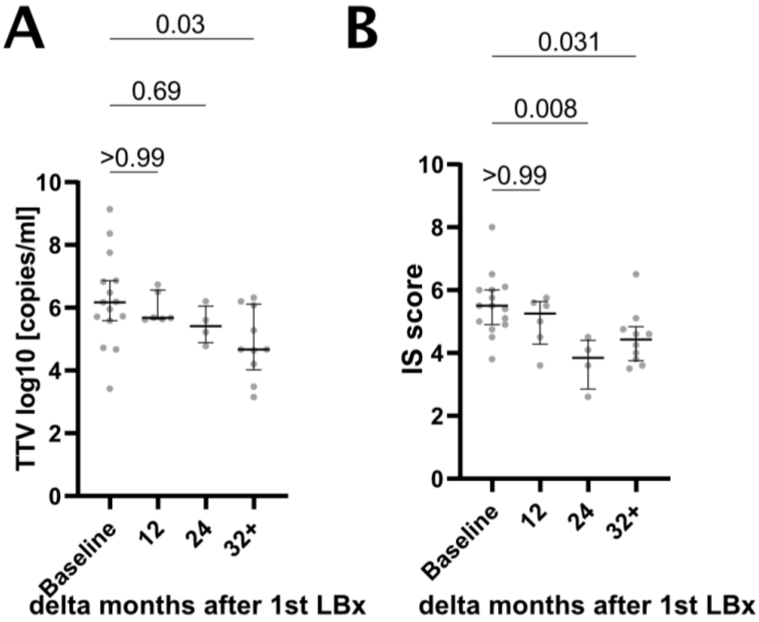

Suppl. Figure 3: Decline of TTV replication and immunosuppression strength over time in longitudinal samples

Patients with follow-up samples were included in this analysis. Magnitude of TTVv (A) and strength of immunosuppression as quantified by IS score (B) correlated with delta time in months from the initial biopsy (Baseline). Stratification was as follows: 12 – up to 12 months between initial liver biopsy (LBx) and follow-up LBx; 24: 13 to 24 months between initial and follow-up LBx; 32+ - at least 32 months between initial and follow-up LBx. Kruskal Wallis Test with Dunn’s post-hoc Test was used for comparison between more than two categorical variables.

Supplemental Figure 4

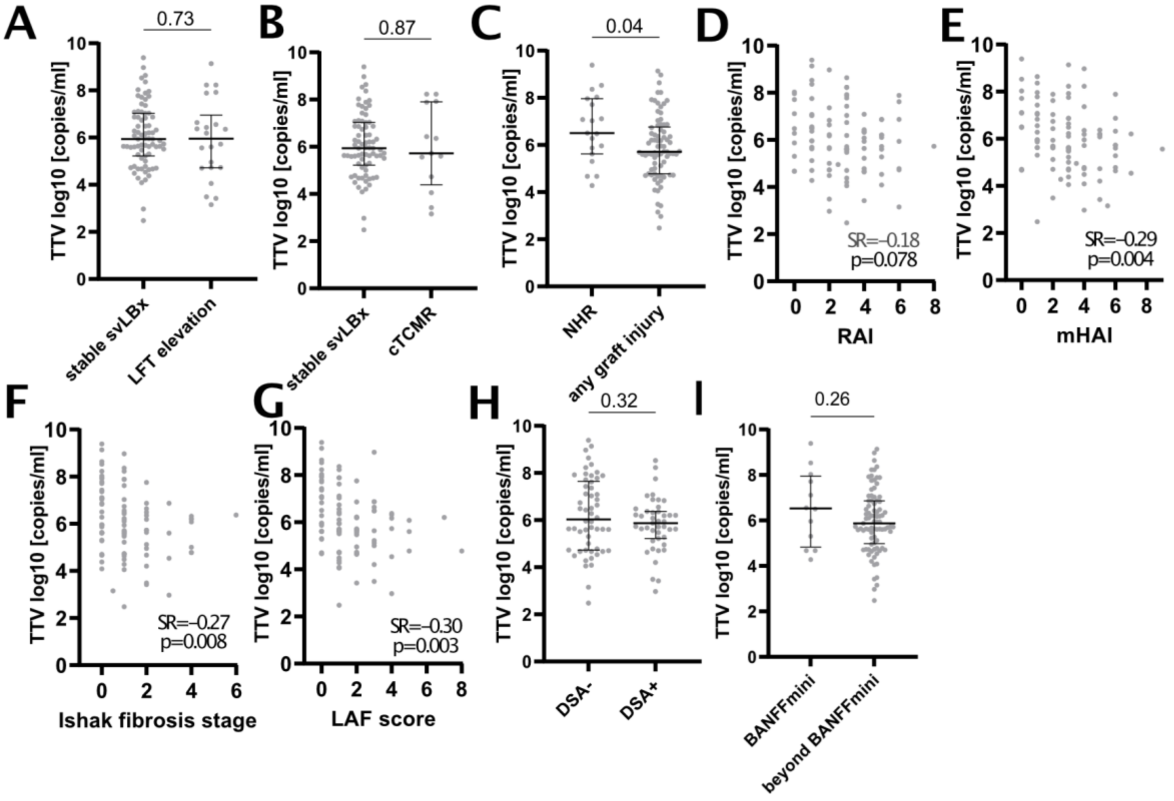

Suppl. Figure 4: Association of TTVv with graft injury and donor specific antibodies

TTVv was not different between patients without elevated liver function tests < 2x ULN (stable svLBx) and with elevated liver function tests (LFT elevation) (A) or patients with clinically overt T cell mediated rejection (cTCMR) (B). TTVv was different between patients with no histological signs of rejection (NHR) and those with any graft injury (C). TTVv correlated with modified histological activity index (mHAI), Ishak fibrosis stage and liver allograft fibrosis (LAF) score but not with rejection activity index (RAI) (D-G). TTVv was not different dependent on the presence of donor specific antibodies (DSA) (H). TTVv was not different dependent on the fulfillment of BANFF criteria for the reduction of immunosuppression (BANFFmini) (I). Spearman rank correlation coefficient (SR) with its respective p values is outlined (D-G). Median and IQR are shown for categorical variables (A-C, H, I). Mann-Whitney U test was used for comparison between two categorical variables.

Supplemental Figure 5

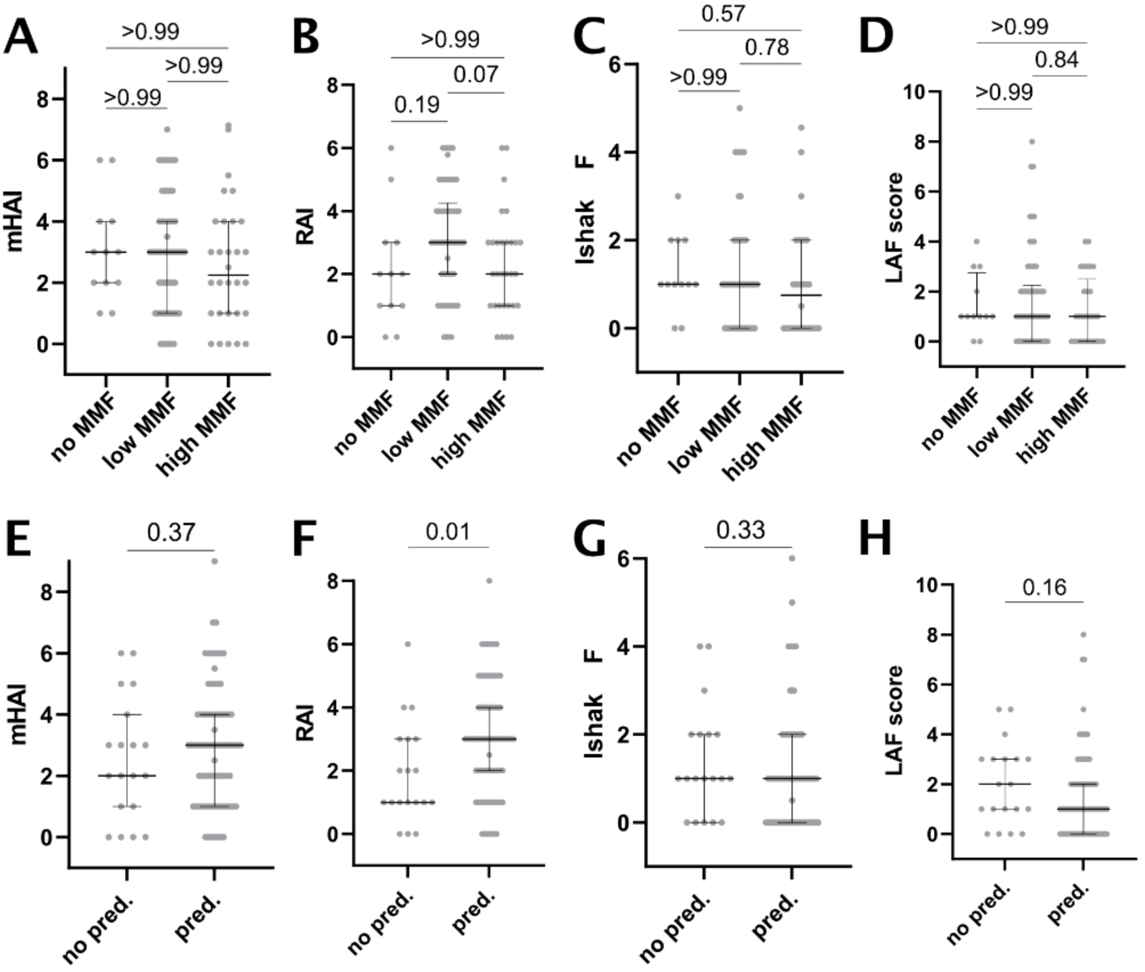

Suppl. Figure 5: Association of TTVv with MMF and prednisolone

mHAI (A), RAI (B), Ishak F (C) and LAF score (D) do not correlate with dosage of mycophenolate mofetil (MMF) (low MMF ≤ 1g/d; high MMF > 1g/d). mHAI (E), Ishak fibrosis stage (G) and LAF score (H) were not different dependent on usage of prednisolone. RAI was higher in patients with prednisolone (F). Median and IQR are shown. The Kruskal-Wallis test with Dunn’s post hoc test was used for comparison between more than two groups and the Mann-Whitney U test was used for the comparison between two groups.

Supplemental Figure 6

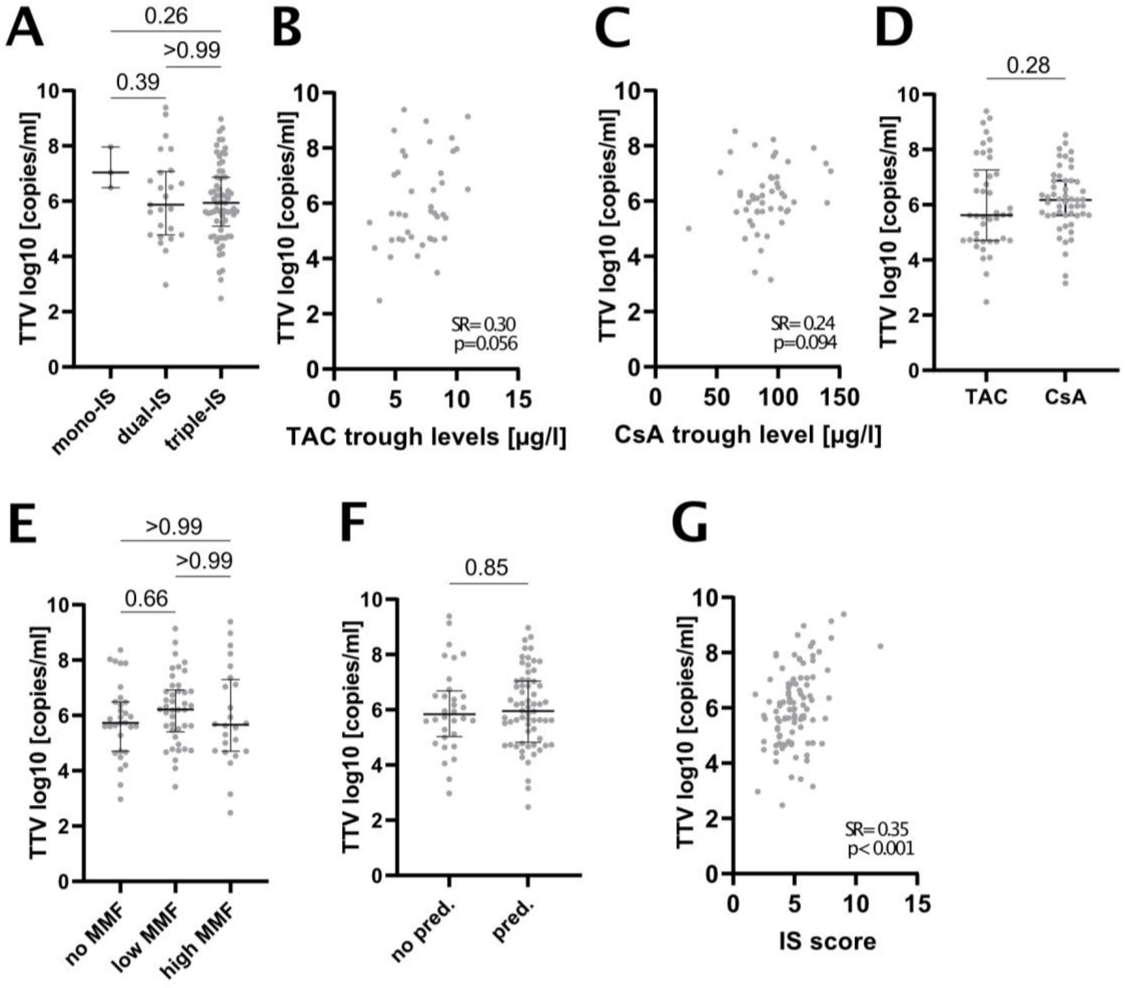

Supplemental Figure 6: Association of TTVv with degree and type of immunosuppression.

Magnitude of TTVv is not different dependent on the number of immunosuppressive drugs used. Mono/dual/triple-immunosuppression (IS): one, two or three immunosuppressive drugs used respectively (A). (B) TTVv does neither correlate with tacrolimus (TAC) (B) and cyclosporine A (CsA) (C). Patient stratification based on the primary immunosuppressive agent used (D). TTVv does not correlate with dosage of mycophenolate mofetil (MMF) (low MMF ≤ 1g/d; high MMF > 1g/d) (E). TTVv does not correlate with usage of prednisolone (pred.) (F). TTVv correlates with IS score (G). Spearman rank correlation coefficient (SR) with its respective p values is outlined (B, C, G). Median and IQR are shown for categorical variables (A, D, E, F). Kruskal Wallis Test with Dunn’s post-hoc Test was used for comparison between

more than two categorical variables, Mann-Whitney U test was used for comparison between two categorical variables.

Supplemental table 1: Demographics of patients stratified according to time after LT

	YEAR 1	> YEAR 1	P
PATIENT NUMBER	47	50	
TTV (LOG10) [COPIES/ML] (MEDIAN (RANGE))	6.87 (3.42 - 9.4)	5.29 (2.5 - 7.9)	< 0.001
AGE [YEARS] (MEDIAN (RANGE))	50 (21 - 69)	52 (18 - 67)	0.63
FEMALE SEX (N (%))	18 (38.3)	17 (34.0)	0.68
TIME AFTER LT [MONTHS] (MEDIAN (RANGE))	7 (2 - 12)	43 (15 - 298)	0.001
SURVEILLANCE LBX (N (%))	36 (76.6)	39 (78.0)	1.00
INDICATION LBX (N (%))	11 (23.4)	11 (22.0)	1.00
LABORATORY PARAMETERS			
AST [TIMES UPPER LIMIT OF NORMAL] (MEDIAN (RANGE))	0.8 (0.4 - 7.2)	0.9 (0.3 - 4.5)	0.78
ALT [TIMES UPPER LIMIT OF NORMAL] (MEDIAN (RANGE))	0.6 (0.1 - 11.8)	0.5 (0.2 - 5.6)	0.50
AP [TIMES UPPER LIMIT OF NORMAL] (MEDIAN (RANGE))	0.9 (0.2 - 8.0)	0.9 (0.3 - 5.0)	0.96
GGT [TIMES UPPER LIMIT OF NORMAL] (MEDIAN (RANGE))	0.8 (0.2 - 38.7)	1.4 (0.2 - 24.4)	0.50
BILIRUBIN [TIMES UPPER LIMIT OF NORMAL] (MEDIAN (RANGE))	0.5 (0.2 - 2.7)	0.6 (0.2 - 4.3)	0.65
PLATELETS [/NL] (MEDIAN (RANGE))	178 (70 - 656)	175 (43 - 394)	0.60
CREATININE [µMOL/L] (MEDIAN (RANGE))	95 (57 - 385)	94 (58 - 792)	0.96
DSA POSITIVE (N (%))	18 (38.3)	25 (50.0)	0.31
HISTOPATHOLOGICAL CHARACTERISTICS			
CTCMR (N (%))	8 (17.0)	5 (10.0)	0.38
SUBTCMR (N (%))	16 (34.0)	23 (46.0)	0.30
IND (N (%))	8 (17.0)	18 (36.0)	0.04
NHR (N (%))	15 (31.9)	4 (8.0)	0.004
RAI (MEDIAN (RANGE))	3 (0 - 8)	3 (0 - 6)	0.15
MHAI (MEDIAN (RANGE))	2 (0 - 6)	3 (0 - 9)	0.002
ISHAK FIBROSIS STAGE (MEDIAN (RANGE))	1 (0 - 3)	1 (0 - 6)	0.003
LAF SCORE (MEDIAN (RANGE))	1 (0 - 4)	2 (0 - 8)	< 0.001
REASON FOR LT			
AILD (N (%))	12 (25.5)	20 (40.0)	0.14
ALCOHOLIC (N (%))	10 (21.3)	10 (20.0)	1.00
HCC (N (%))	8 (17.0)	4 (8.0)	0.22
VIRAL (N (%))	3 (6.4)	1 (2.0)	0.35
CRYPTOGENIC (N (%))	6 (12.8)	6 (12.0)	1.00
OTHER (N (%))	8 (17.0)	9 (18.0)	1.00
IMMUNOSUPPRESSION			
MONO-IS (N (%))	2 (4.3)	1 (2.0)	0.61
DUAL-IS (N (%))	10 (21.3)	15 (30.0)	0.36
TRIPLE-IS (N (%))	35 (74.5)	34 (68.0)	0.51

**TAC (N (%))**	22 (46.8)	20 (40.0)	0.54
**CSA (N (%))**	23 (48.9)	28 (56.0)	0.55
**EVR (N (%))**	2 (4.3)	0 (0.0)	0.24
**SIR (N (%))**	1 (2.1)	1 (2.0)	1.00
**IS SCORE (MEDIAN (RANGE))**	5.5 (3 - 12)	4.5 (1.75 - 7.5)	< 0.001

TTV: torque teno virus; AST: aspartate aminotransferase; ALT: alanine aminotransferase; AP: alkaline phosphatase; gGT: gamma-glutamyltransferase; cTCMR: clinically overt T-cell mediated rejection; subTCMR: subclinial TCMR; IND: indeterminate graft injury other than cTCMR and subTCMR; NHR: no histological signs of rejection; RAI: rejection activity index; mHAI: modified histological activity index according to Ishak et al[21]; LAF score: liver allograft fibrosis score according to Venturi et al.[24]; DSA: donor specific anti-HLA antibodies; LT: liver transplantation; CLD: chronic liver disease; AILD: autoimmune liver disease; HCC: hepatocellular carcinoma; IS: immunosuppression; TAC: tacrolimus; CsA: cyclosporine A; EVR: everolimus; SIR: sirolimus; IS score: immunosuppression score.

Supplemental Table 2: Correlation of pharmacokinetic markers of immunosuppression degree with histological scores of graft injury

	mHAI	RAI	Ishak F	LAF score
IS score	SR = -0.18 (p = 0.07, n = 97)	SR = -0.13 (p = 0.22, n = 97)	SR = -0.34 (p = 0.001, n = 97)	SR = -0.35 (p < 0.001, n = 96)
TAC trough level [µg/l]	SR = 0.09 (p = 0.59, n = 42)	SR = 0.03 (p = 0.84, n = 42)	SR = 0.032 (p = 0.84, n = 42)	SR = 0.17 (p = 0.30, n = 42)
CsA trough level [µg/l]	SR = -0.13 (p = 0.37, n = 51)	SR = -0.12 (p = 0.39, n = 51)	SR = -0.12 (p = 0.41, n = 51)	SR = -0.08 (p = 0.58, n = 50)

Correlation matrix between markers to quantify degree of immunosuppression (IS score, TAC trough level and CsA trough level) and histological parameters of graft injury (mHAI, RAI, Ishak F and LAF Score). Values are provided as Spearman correlation coefficient (SR). P-value and number (n) are shown in bracket. RAI: rejection activity index; mHAI: modified histological activity index according to Ishak et al[21]; Ishak F: fibrosis staging according to Ishak et al[21]; LAF score: liver allograft fibrosis score according to Venturi et al.[24]; TAC: tacrolimus; CsA: cyclosporine A. Mann-Whitney U test was used for group comparisons between continuous variables and Fisher's Exact test for group comparisons between caterogical variables. P-values below 0.05 were regarded as significant.
